# miR-148b reverses cisplatin-resistance in non-small cell cancer cells via negatively regulating DNA (cytosine-5)-methyltransferase 1(DNMT1) expression

**DOI:** 10.1186/s12967-015-0488-y

**Published:** 2015-04-28

**Authors:** Chengguang Sui, Fandong Meng, Yan Li, Youhong Jiang

**Affiliations:** Molecular Oncology Department of Cancer Research institution, The First Hospital of China Medical University, 155 Nanjing North Street, He-ping District of Shenyang City, Liaoning Province 110001 China

**Keywords:** DNA (cytosine-5)-methyltransferase 1(DNMT1), Cisplatin resistance, MicroRNA-148b, A549 cell, SPC-A1 cell

## Abstract

**Background:**

The emergence of drug resistance in cancer patients limits the success rate of clinical chemotherapy. MicroRNAs (miRNAs) may play a role in chemoresistance and may be involved in modulating of some drug resistance-related pathways in cancer cells. In this study, the involvement of microRNA-148b (miR-148b) and its roles in the development of chemoresistance in lung cancer are determined.

**Methods:**

This study was performed in two lung cancer cell lines (A549 and SPC-A1). The levels of miR-148b and DNMT1 mRNA expression were determined by using Quantitative Real-Time PCR. Proteins of DNMTs are represented by western blot assay. Cell viability was assessed by MTT assay. Cell apoptosis was evaluated using flow cytometry.

**Results:**

The data showed a down-regulated of miR-148b expression and evaluated methyltransferases (DNMTs) expression in cisplatin-resisted human non-small cell lung cancer (NSCLC) cell line-A549/DDP and SPC-A1/DDP compared with their parental A549 and SPC-A1 cell line. In transfection experiments, miR-148b mimics reduced the DNMT1 expression, as well as enhanced the sensitivity of cells to cisplatin and cisplatin-induced apoptosis in A549/DDP or SPC-A1/DDP cells. While miR-148b inhibitor increased DNMT1 expression, as well as attenuated the sensitivity of cells to cisplatin in A549 and SPC-A1 cells. miR-148b was showed to exert negative effect on DNMT1 expression by targeting its 3′UTR in A549/DDP and A549 cells. Importantly, silenced DNMT1 increases cisplatin sensitivity of A549/DDP cells and over-expressed DNMT1 reverses pro-apoptosis effect of miR-148b mimic.

**Conclusions:**

miR-148b reverses cisplatin-resistance in non-small cell cancer cells via negatively regulating DNMT1 expression.

## Background

Lung cancer is regarded as the leading cause of cancer related deaths worldwide [1]. Among this, non-small cell lung cancer (NSCLC), which distinguished to small-cell lung cancer (SCLC) from pathological and histological characteristics, represents approximately 85% [2] and its five-year survival is only 15% [3]. Chemotherapy is well known as the main method to treat lung cancer in earlier stages of treatment, especially as adjuvant chemotherapy after surgery. Although advances in cisplatin-based chemotherapy have resulted in improving the rate of survival, their therapeutic efficacy was limited for the development of cisplatin resistance. Therefore, a good understanding of the molecular mechanisms underlying cisplatin resistance development is urgently needed.

microRNAs are small non-coding RNA molecules consisting of 20–24 nucleotides and function as the suppressor for gene expression by interacting with the 3′-untranslated regions (3′UTRs) of target mRNAs at 5–7 nucleotides. These interactions may lead to either obstruction of translation or degradation of the targeted mRNAs [4]. Dysregulation of miRNAs in cells may result in alterations in cellular differentiation, proliferation, apoptosis and metastasis processes that are important in the development of cancer [5]. Recently, accumulated studies have shown that miRNAs may play a role in chemoresistance of cancer treatment and may be involved in the modulation of some drug resistance-related pathways in some cancer cells.

Although few studied focus on the involvement of microRNA-148b (miR-148b) in drug resistance-related miRNAs, it has been described to be down-regulated in several types of cancers including pancreatic cancer [6,7] colorectal cancer [8], gastric cancer [9], and basal-line breast cancer [10]. Furthermore, recent report indicates that miR-148b acts as a tumor suppressor by targeting specific oncogenes in NSCLC cells [11]. Importantly, miR-148a, which is together with miR-148b belonging to miR-148/152 family, plays an important role in improving response to chemotherapy in sensitive and resistant cancers. DNA methyltransferases (DNMTs), functioned as an important regulator for epigenetic processes of chemotherapy [12], have been proved to be regulated by miR-148b [7]. Thus, we hypothesized that miR-148b might be involved in chemotherapy resistance of NSCLC.

In this study, we focused on the effect of altered miR-148b expression on cisplatin resistance of A549/DDP and SPC-A1/DPP cells. We also investigated the possible targets for miR-148b. This study will help to better understand the biological activities of miR-148b in NSCLC treatment.

## Methods

### Cells culture and transfection

Human lung adenocarcinoma cell line (A549 and SPC-A1) and their cisplatin-resistant variant A549/DDP and SPC-A1/DDP (purchased from the Academy of Military Medical Science, Beijing, P.R. China.) were cultured in RPMI-1640 medium (Invitrogen, Carlsbad, CA, USA) supplemented with 10% fetal calf serum (Gibco), penicillin (100 μg/mL), and streptomycin (100 mg/mL) in a humidified atmosphere containing 5% CO2 at 37°C. In order to maintain cisplatin-resistant phenotype, A549/DDP and SPC-A1/DDP cells were maintained in the medium additionally contained 2 μg/mL cisplatin (DDP).

The cells were seeded in 6-well plates at 1 × 10^5^ cells/well followed by cultured for 24 hours and then transfected with 25 nmol of miR-148b mimics and negative control mimics (NC), miR-148b inhibitors (anti-miR-148b) and negative control inhibitors (anti-NC) (RIBO Bio, Guangzhou, P.R. China) using Lipofectamine 2000 (Invitrogen), according to the manufacturer’s protocol. The effect of mimics or antagomirs of miR-148b was examined in triplicate at 24 h post-transfection. DNMT1 gene was knocked down using DNMT1 interfering small RNA (siRNA), which was obtained from Generay (Generay, Shanghai, China) and transfected into the A549/DDP cells by Lipofectamine 2000.

### Quantitative REAL-TIME PCR

Total RNA was extracted from the cells with the Trizol Reagent (Invitrogen, Carlsbad, CA, USA). Quantitative Real-Time PCR (Q-RT-PCR) assays was to quantify the mature miR-148a and DNMT1 mRNA using fluorescent nucleic acid dye. Each sample (1 μg) was reverse-transcribed into cDNA by using the RevertAidTM First Strand cDNA Synthesis Kit (Fermentas). Real-time PCR was performed in the Applied Biosystems 7500 Real-time PCR system using SYBR Premix ExTagTM (Takara) according to the manufacturer's protocols. All reactions were run in triplicate. The threshold cycle (CT) is defined as the fractional cycle number at which the fluorescence passes the fixed threshold. The miR-148b expression levels were normalized to U6 RNA. And the DNMT1 mRNA was normalized to β-actin mRNA. The relative expression was calculated using the comparative CT method.

### 3-(4,5-dimethylthiazol-2-yl)-2,5- diphenyltetrazolium bromide (MTT) Assay

The cells separately were seeded in 96-well plates at a density of 4 × 103 cells/well in culture medium. After their adherence to the culture dish, cells were incubated with mimic or inhibitor for miR148b for 24 hours. And then A549/DDP cells were treated with cisplatin at a series of concentrations (10, 20, 40, 80, 160 and 320 μg/mL), while A549 cells were treated with cisplatin at 2, 4, 8, 16, 32 and 64 μg/mL. Next, cells were cultured for an additional 48 hours and cell survival was measured by MTT assay according to the manufacturer’s instructions. The resulting absorbance at 490 nm was measured on a spectrophotometer. The concentration at which drug produced 50% inhibition of growth (IC50) was estimated by the relative survival curve.

### Western blot

The cells were lyzed in RIPA buffer in the presence of proteinase inhibitor (Sigma-Aldrich, St. Louis, MO, USA). Equal amounts of protein was separated on 10% sodium dedecyl sulfate polyacrylamide gel electrophoresis (SDS-PAGE) and transferred to PVDF membrane (Millipore Corp, Billerica, MA, USA). Membranes were blocked with 10% skimmed milk followed by incubation with the antibodies (1:500): anti-DNMT1(sc-10222; Santa Cruz, CA, USA), anti-DNMT3a (sc-20703; Santa Cruz, CA, USA), anti-DNMT3b ( sc-10236; Santa Cruz, CA, USA) and anti-β-Actin (#12620; Cell Signaling Technology, MA, USA). After washed extensively with 0.1% in phosphate-buffered saline (PBS), the membranes were incubated with secondary antibodies (anti-rabbit, 1:1000) conjugated with horseradish peroxidase. The reaction was detected with enhanced chemiluminescence. Protein levels were normalized to β-Actin.

### Flow cytometry-based apoptosis

The A549/DDP cells were seeded in 96-well plates at a density of 4 × 103 cells/well in culture medium and transfected. Twenty-four hours later, 10 μg/mL cisplatin was added to the medium and the cells were incubated for 48 hours. After incubation, the cells were harvested, stained with 5 μL annexin V-flourescein isothiocyanate (FITC) and 10 μL propidium iodide (PI) (20 μg/mL). The mixture was incubated at room temperature in the dark for 15 minutes and analyzed by fluorescence-activated cell sorting (FACS).

### Bioinformatics analysis of miR-148b target in DNMT1

**Figure 1:**
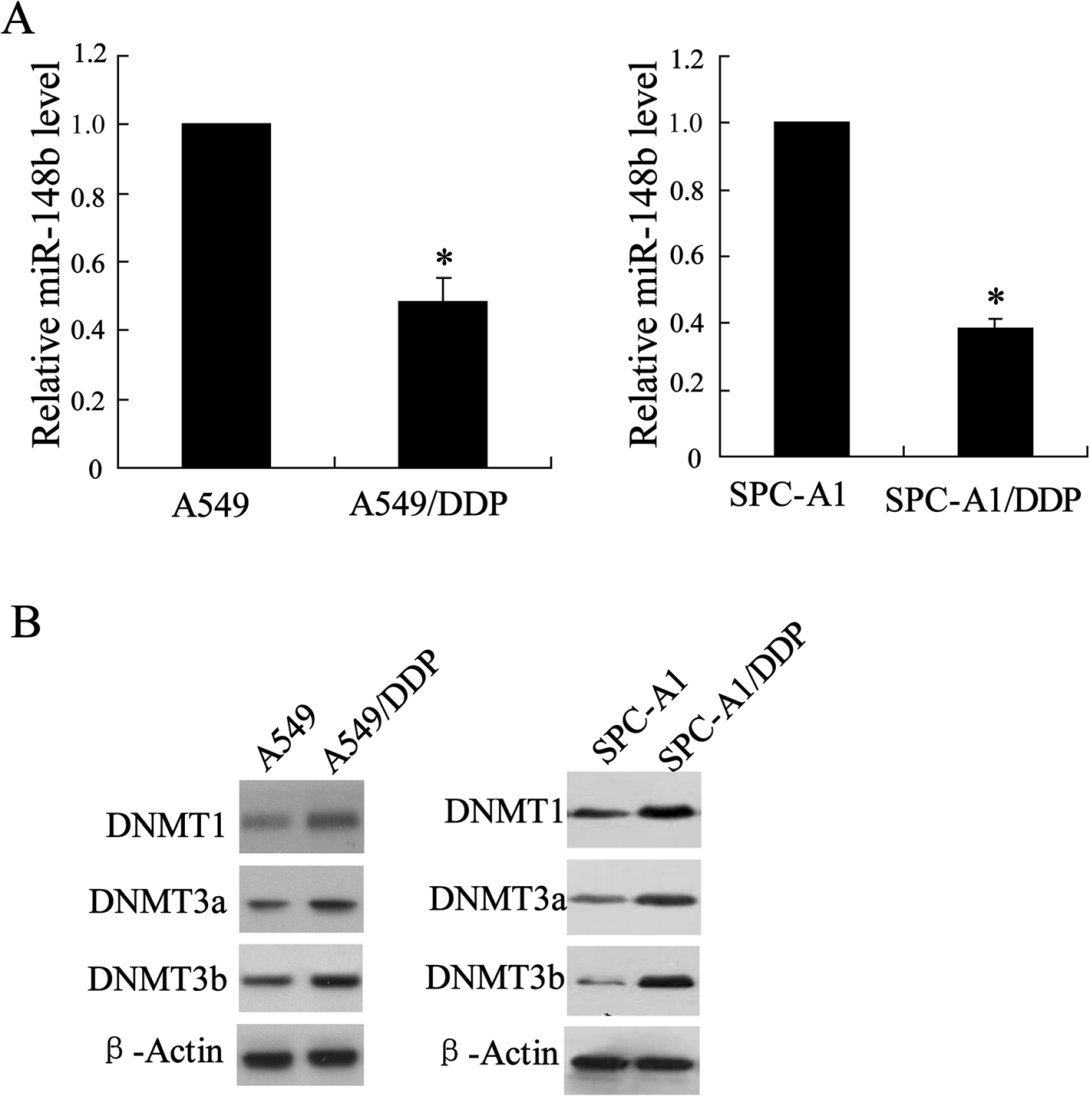
Expression of miR-148b and DNMTs in two cell lines of lung cancer. Cells were lyzed to detect level of **(A)** miR-148b using Q-RT-PCR and **(B)** NDMT1, NDMT3a and NDMT3b using western blot assay in A549/DDP, SPC-A1/DDP and their respective parental cells. Data were represented as mean ± SD. *P <0.05 compared with A549.

Based on bioinformatics analysis, we predicted that hsa-miR-148b can bind with the 3′UTR region of NADM1 by using four common websites (Target Scan: http://www.targetscan.org/, miRanda: http://www.microrna.org/, Microcosm: http://www.ebi.ac.uk/enright-srv/microcosm/cgi-bin/targets/v5/genome.pl, and PITA: http://genie.weizmann.ac.il/) (Figure 1A).

### Plasmid construction and Luciferase reporter assays

For DNMT1 3′UTR reporter assay, cells were placed in 24-well plates (1 × 105 cells per well) and then cotransfected with pGL3-DROSHA 3′UTR-T or pGL3-DROSHA 3′UTR-C and pRL-SV40 (50:1). The mimics and inhibitors of hsa-miR-148b and their negative controls (RIBO Bio, Guangzhou, P.R. China) were cotransfected with the reporter plasmids at a final concentration of 20 nmol/μl. 48 hours after transfection in A549/DDP and A549 cells, luciferase activity in lysates was measured with a Dual-Luciferase Reporter Assay System (Promega, WI, USA) and normalized against the activity of the pRL-SV40. The assays were conducted followed by the manufacture’s suggestions. Independent triplicate experiments were performed for each plasmid construct.

### Statistical analysis

All experiments were run in triplicate. All statistical analysis were performed using SPSS 13.0. Data were expressed as means ± standard deviation (SD). The difference between the groups was analyzed using Student’s t test when only two groups were compared or one-way analysis of variance (ANOVA) when more than two groups were compared. Values of *P* < 0.05 indicate statistical significance.

## Results

### Differential expression of miR-148b and DNMTs in both A549/DPP and SPC-A1/DPP cells compared with their parental cell lines

**Figure 2:**
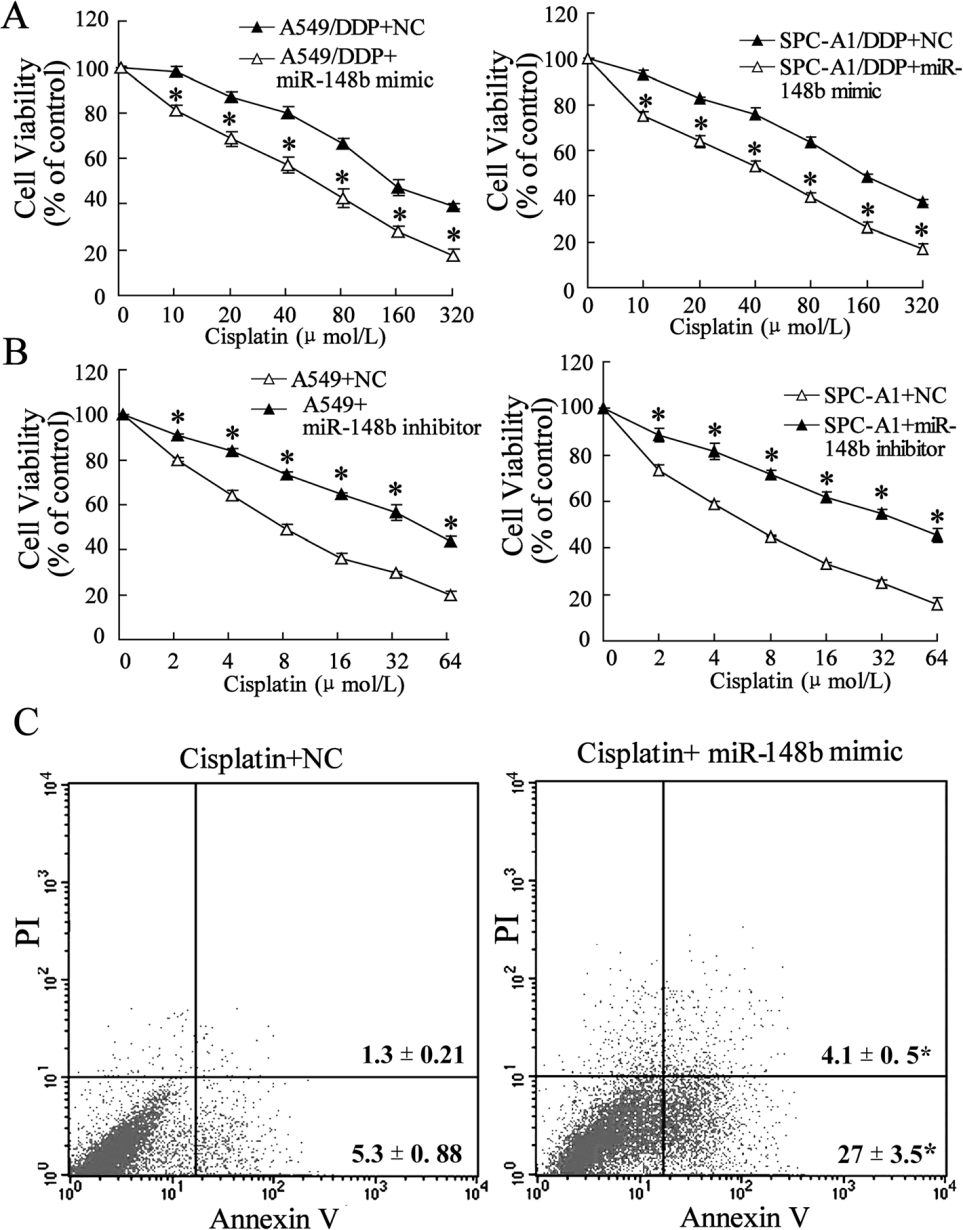
miR-148a modified the cisplatin sensitivity of A549/DDP and SPC-A1/DDP cells. **(A)** After A549/DDP and SPC-A1/DDP cells transfected by miR-148b mimic or NC for 24 h, the cells were incubated with various doses of cisplatin (10, 20, 40, 80, 160, 320 μmol/L) for 48 h , and then cells viability was assessed by MTT assay. In A549/DDP cell, IC50miR-148b mimic is 55.36 μmol/L, IC50NC is 173.27 μmol/L; in SPC-A1/DDP cell, IC50miR-148b mimic is 44.25 μmol/L, IC50NC is 156.32 μmol/L. **(B)** After A549 and SPC-A1 cells transfected by miR-148b inhibitor or NC for 24 h, the cells were incubated with various doses of cisplatin (2, 4, 8, 16, 32 μmol/L) for 48 h, and then cells viability was assessed by MTT assay. In A549 cell, IC50miR-148b inhibitor is 45.06 μmol/L, IC50NC is 9.21 μmol/L; in SPC-A1 cell, IC50miR-148b inhibitor is 44.62 μmol/L, IC50NC is 6.97 μmol/L. **(C)** After A549/DDP cells treated by NC or miR-148b mimic for 24 h, and incubated with 10 μmol/L cisplatin for 48 h, cells apoptosis was evaluated using flow cytometry. Data were represented as mean ± SD. *P <0.05 compared with cells treated with the same concentration of ciaplatin accordingly.

We detected the expression levels of miR-148b and DNMTs protein in A549/DPP and SPC-A1/DPP as well as their parental cell lines using Q-RT-PCR and Western blot, respectively. The results showed that miR-148b had an average of 2-fold lower expression level in A549/DPP cells and an average of 2.5-fold lower expression level in SPC-A1/DPP cells than that in A549 cells and SPC-A1 cell, respectively (*P* < 0.05, Figure 2A). On the contrary, the expression of DNMT1, DNMT3a and DNMT3b was upregulated in A549/DPP and SPC-A1/DPP cell lines (Figure 2B).

### Upregulation of miR-148b increased sensitivity of the A549/DDP and SPC-A1/DPP cells to cisplatin

**Figure 3:**
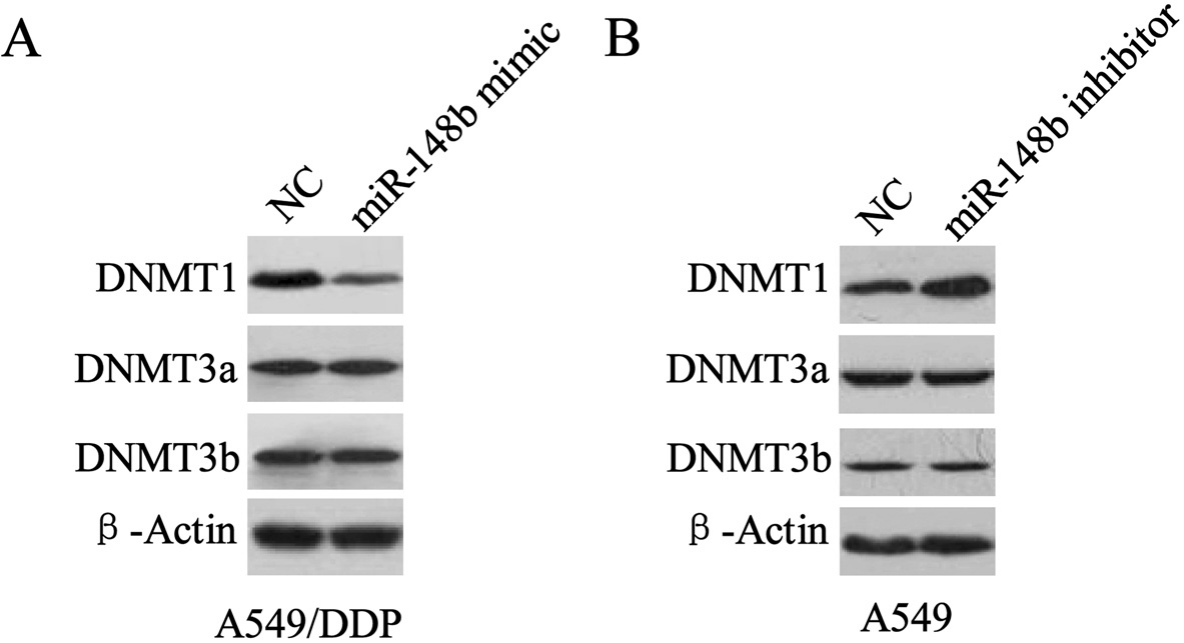
miR-148a modified expression of DNMTs. A549/DDP and A-549 cells were separately transfected by NC or miR-148b mimic or miR-148b ainhibitor for 24 h. Expressions of DNMT1, DNMT3a and DNMT3b in cells were determined using western blot assay in **(A)** A549/DDP cells and **(B)** A549 cells.

To investigate whether miR-148b could modulate the sensitivity of the A549/DPP and SPC-A1/DPP as well as their parental cell lines to cisplatin, we transfected cisplatin-resistant cells with mimics of miR-148b and their parental cells with inhibitors of miR-148b, respectively, and then the cells were treated with a series of concentrations of cisplatin. We got the data that the mimics of miR-148b increased the sensitivity to cisplatin significantly by 1.5-fold in A549/DDP and SPC-A1/DPP cells (*P* < 0.05, respectively; Figure 3A), however, sensitivities of A549 and SPC-A1 cells transfected with miR-148b inhibitor to cisplatin were decreased compared with that treated with NC (*P* < 0.05, respectively; Figure 3B). To further assess the role of miR-148b in regulating growth of A549/DDP and SPC-A1/DPP cells exposed to cisplatin, cells apoptosis rate that had been transfected with miR-148b mimic were analyzed using flow cytometry assay. As shown in Figure 3C, compared with NC, miR-148b mimic promoted cisplatin-induced apoptosis of A549/DDP and SPC-A1/DPP cells (*P* < 0.05, respectively).

### MiR-148b regulates expression of DNMT1

**Figure 4:**
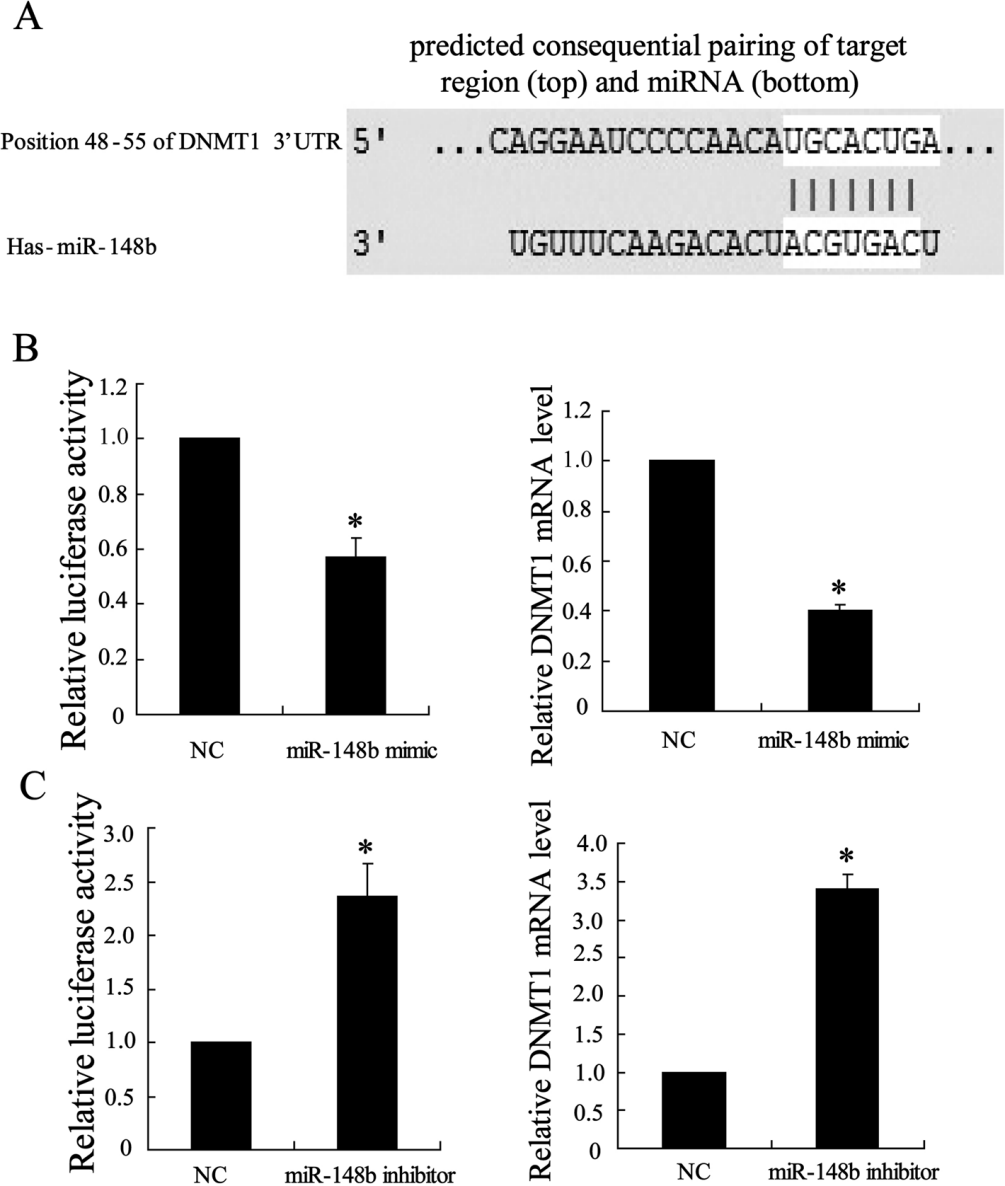
Targeting site of miR-148b in the DNMT1 3′UTR. **(A)** DNMT1 3′UTR was predicted a binding site for hsa-miR-148b. The mouse DNMT1 mRNA sequence is shown with potential binding sites indicated in white. The highly conserved mature miR-148b sequence in mammals and potential binding between the miR-148b seed region to the mouse DNMT1 3′UTR sequence are shown. **(B)** After A549/DDP transfected with miR-148b mimic for 24 h, binding of miR-148b and DNMT1 was assessed by Luciferase reporter assays and expression of DNMT1 mRNA was assessed using Q-RT-PCR. **(C)** After A549 transfected with miR-148b ainhibitor for 24 h, binding of miR-148b and DNMT1 was assessed by Luciferase reporter assays and expression of DNMT1 mRNA was assessed using Q-RT-PCR. Data were represented as mean ± SD. *P <0.05 compared with NC

To determine whether miR-148b is involved in regulation the expression of DNMTs, A549/DDP and A549 cells were transfected with the mimics or inhibitors of miR-148b respectively, and the expression levels of DNMTs were determined by Western blot. Figure 4A showed that transfected mimics of miR-148b resulted in downregulating expression of DNMT1 in A549/DDP cells, but mimics of miR-148b exerted no effect on expression of DNMT3a and DNMT3b. In addition, expression of DNMT1 in A549 transfected with miR-148b inhibitor was upregulated, however, expressions of DNMT3a and DNMT3b were not changed (Figure 4B).

### Targeting site of miR-148 in the DNMT1 3′UTR

DNMT1 was regarded as a potential target gene of miR-148b using TargetScan Release 5.2 in which we found a binding site for miR-148b in the 3′UTR of DNMT1 mRNA (Figure 1A). To confirm DNMT1 as a real miR-148b target, the entire 3′UTR of DNMT1 was inserted downstream of the luciferase gene and assayed in A549/DDP and A549 cells. As shown in Figure 1B, cotransfection of miR-148b mimics with the DNMT1 3′UTR reporter resulted in a decrease (40%) in luciferase activity and a highly significant decline in mRNA of DNMT1 in A549/DDP cells. Additionally, Figure 1C showed increases (2.3 fold) in luciferase activity and mRNA of DNMT1 in A549 transfected with miR-148b inhibitor and the DNMT1 3′UTR reporter. These data suggested a might involvement of DNMT1 in modulating the cisplatin sensitivity by miR-148b in the A549/DPP and A549 cells.

### DNMT1 siRNA increases cisplatin sensitivity of A549/DDP and SPC-A1/DPP cells

**Figure 5:**
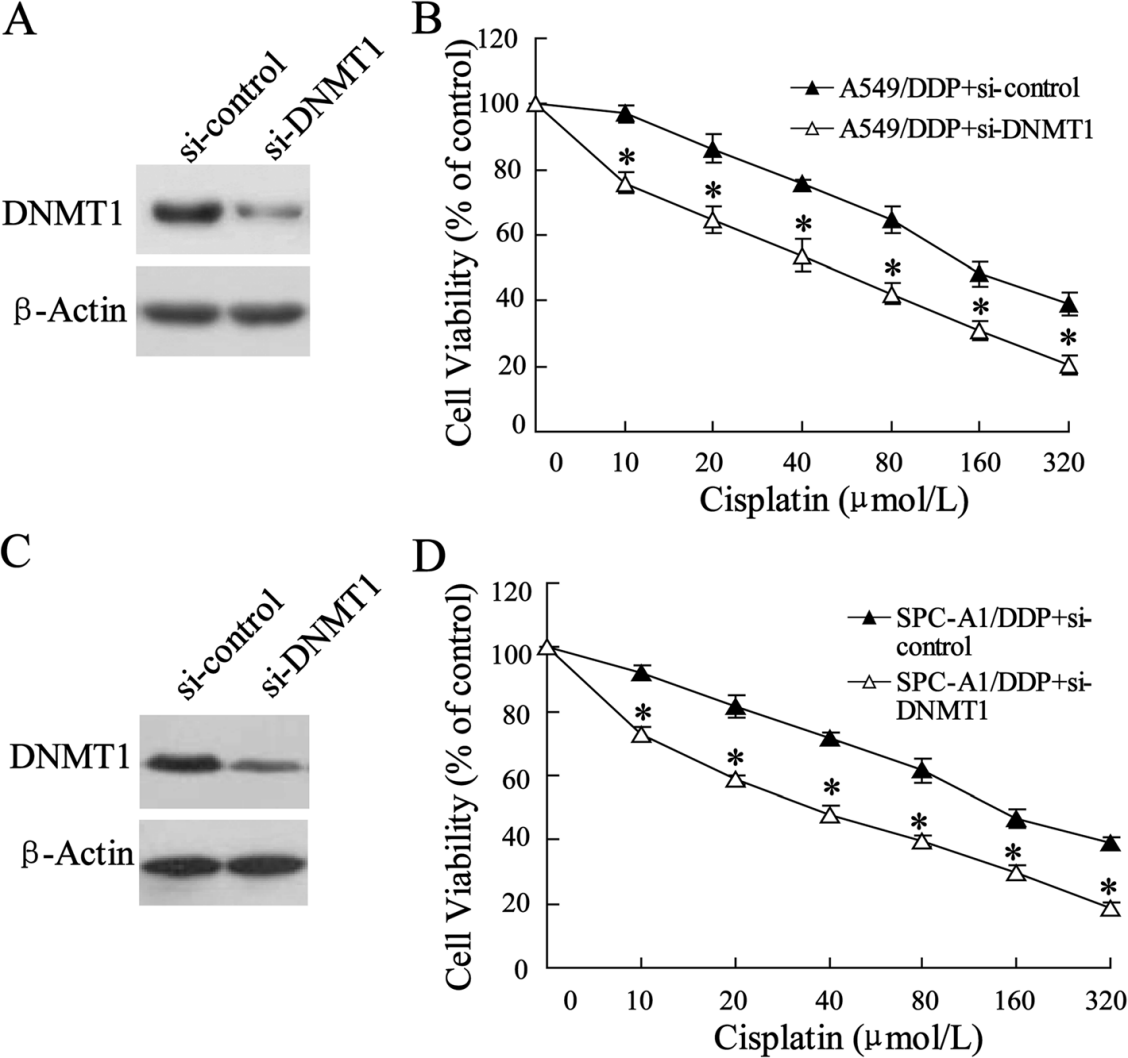
Effect of downregulated DNMT1 on cisplatin sensitivity of A549/DDP and SPC-A1/DDP cells. **(A)** A549/DDP cells were transfected with si-DNMT1. **(B)** 24 hours after incubation, the cell viability was assessed by MTT assay. **(C)** SPC-A1/DDP cells were also transfected with si-DNMT1 and **(D)** its cell viability was evaluated. Data were represented as mean ± SD. *P <0.05 compared with cells treated with the same concentration of ciaplatin accordingly.

To demonstrate the effect of DNMT1 on cisplatin sensitivity of lung cancer cells, the DNMT1 siRNA or siRNA negative control was transfected into the A549/DDP and SPC-A1/DPP cells to observe cell viability. We first measured the expression of DNMT1 using western blot and the data indicated a declining level of DNMT1 in A549/DDP cell (Figure 5A) and SPC-A1/DPP cell (Figure 5C). To further investigate the effect of DNMT1 on cisplatin-resistant cell chemoresistance, we analyzed the sensitivity of A549/DDP and SPC-A1/DPP cells to a series concentrations of ciaplatin after knock-down of DNMT1. The growth-inhibitory activities of ciaplatin in the A549/DDP-DNMT1 and SPC-A1/DPP-DNMT1 were 1.2-fold lower than that in control cells (*P* < 0.05, repectively; Figure 5B and D). These demonstrate that down-regulation of DNMT1 could reverse the resistance of A549/DDP cells as well as SPC-A1/DPP to ciaplatin.

### Overexpression of DNMT1 reverses miR-148b-increased cisplatin sensitivity of A549/DDP cells

**Figure 6:**
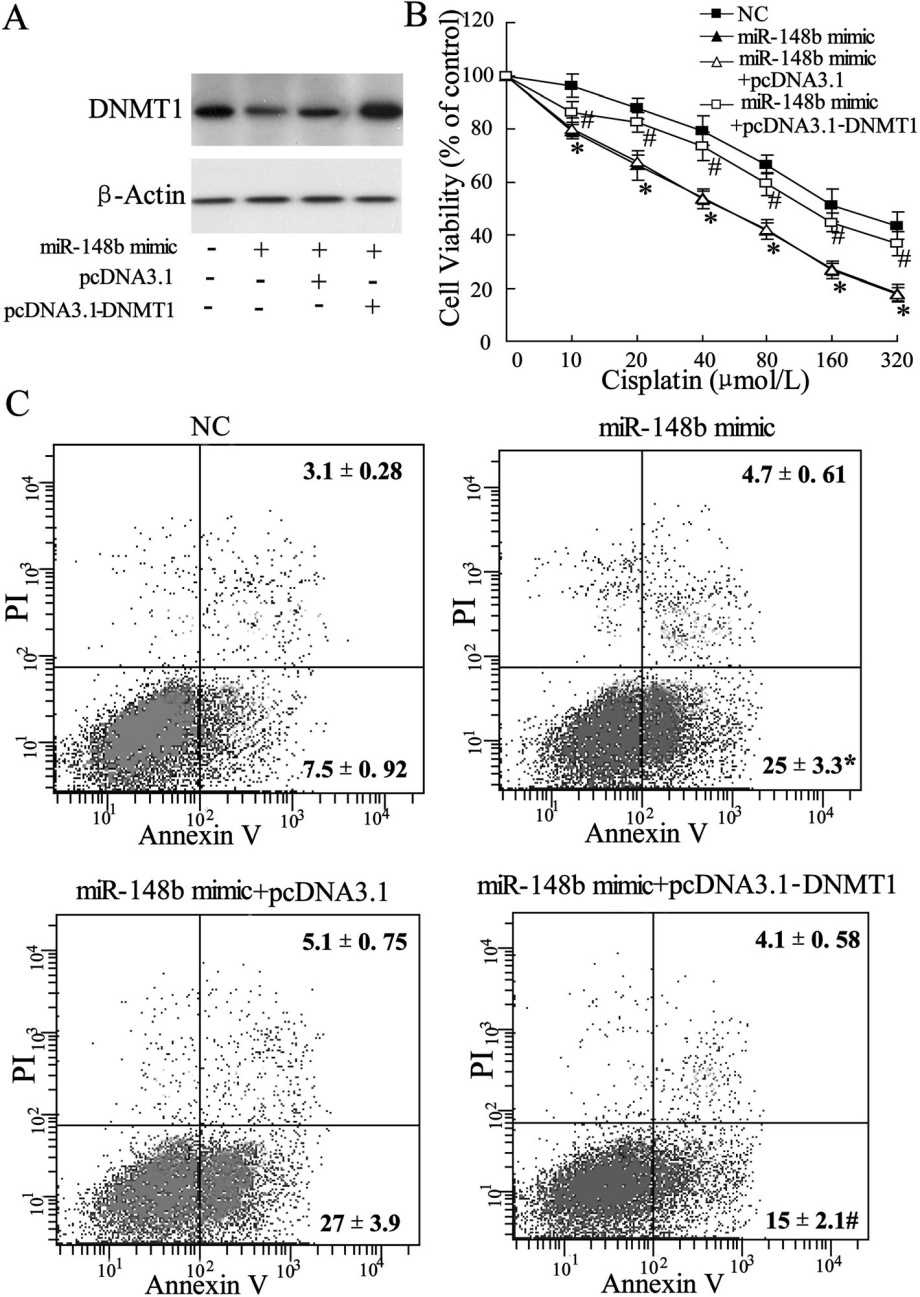
Effect of overexpressed DNMT1 on cisplatin sensitivity of A549/DDP cells. A549/DDP cells transfected with pcDNA3.1-DNMT1 or pcDNA3.1 were used in experiments. **(A)** Expression of DNMT1 was evaluated using western blot assay. **(B)** After transfected with miR-148b mimic for 24 h, cells were incubated with various doses of cisplatin (10, 20, 40, 80, 160, 320 μmol/L), and then cell viability was assessed using MTT assay. **(C)** After transfected with miR-148b mimic24 for 24 h, cells were incubated with 10 μmil/L cisplatin, and then cell apoptosis was determined using flow cytometry. Data were represented as mean ± SD. *P <0.05 compared with NC; #P <0.05 compared with miR-148b mimic.

To conform the regulating role of DNMT1 in miR-148b modulating the cisplatin sensitivity of the A549/DPP cells, the pcDNA3.1-DNMT1 or pcDNA3.1 negative control was transfected into the A549/DDP cells. Western blot assay showed an increased expression of DNMT1 in A549/DDP cells cotransfected with pcDNA3.1-DNMT1 and miR-148b mimics (*P* < 0.05, Figure 6A). Next, cells were treated with a series concentration of ciaplatin to evaluate cells viability by MTT assay. As shown in Figure 6B, compared with miR-148b mimic NC, A549/DDP cells transfected with pcDNA3.1-DNMT1 reversed miR-148b-increased cisplatin sensitivity of A549/DDP cells (*P* < 0.05). Consistent with this data, apoptosis in A549/DDP cells transfected with pcDNA3.1-DNMT1 was lower than that in cells transfected with miR-148b mimic NC (*P* < 0.05, Figure 6C).

## Discussion

Cisplatin-based chemotherapy is the first-line chemotherapy for advanced-stage patients in various cancers at present. Despite a high rate of initial response, 5-year patient survival rate is very low resulting from tumors developing resistance to therapy [13]. Thus, an intense research underlying chemoresistance should be conducted to further establish better therapeutic approaches. In this research, we first detected the impact of miR-148b expression on ciaplatin resistance in NSCLC cells line and the results showed that miR-148b was notably down-regulated in cisplatin-resistant NSCLC cell line (A549/DDP). The further analysis indicated that DNMT1 is the important target for miR-148b in the development of cisplatin resistance in NSCLC cells .

miR-148b is a member of miR-148/152 family, which has the mature structure of 21–22 nucleotides in length and the same seed sequence of approximately 6–7 nucleotides [14]. It has been demonstrated that down-regulated miR-148b is correlated with tumor formation, distant metastasis or worse prognosis in colorectal and gastric cancer [7,8] and it also suppressed proliferation and migration of NSCLC cells [11] However, the study in relationship between miR-148b and chemotherapy resistance is very rare. Interestingly, as a member of miR-148/152 family, miR-148a plays an important role in improving response to chemotherapy in sensitive and resistant cancers, such as oesophageal adenocarcinoma and squamous cell carcinoma cells [15] and hormone-refractory prostate cancer [16]. In this study, we focus on the changes in miR-148b expression and hypothesized its involvement in chemotherapy resistance of NSCLC. Our data showed the down-regulated expression of miR-148b in A549/DDP and SPC-A1/DPP cells compared with their parental cell lines. Importantly, over-expression of miR-148b increased the sensitivity of A549/DDP as well as SPC-A1/DPP cells to cisplatin measured by MTT and flow cytometry assay. Therefore, the results indicated that miR-148b could play a crucial role in the development of cisplatin resistance in NSCLC.

DNMTs is a kind of most abundant DNA methyltransferase in mammalian cells and mediate the transfer of methyl groups from S-adenosylmethionine to the 5 position of cytosine bases in the dinucleotide sequence CpG [17]. DNMTs play an important role in the epigenetic processes that including gene expression and the maintenance of genome integrity. Accumulated evidence has been demonstrated that DNMTs mediated transcriptional silencing in malignant tumors as well as in lung cancer. In this study, we also detected up-regulated DNMT1, DNMT3a and DNMT3b in A549/DDP cells. DNMTs regulation by miR-148/152 family members has been reported in a number of human diseases [18,19]. However, the result in our research indicated that only DNMT1 was regulated by miR-148a. In addition, Xiang et al. reported that DNMT1 is a key target for miR-152 and miR-185 in ovarian cancer cisplatin resistance in vitro and in vivo [20]. DNMT1 was also involved in gastric carcinomas treated by neoadjuvant chemotherapy [21]. These results suggest that DNMT1 is an key target gene for miRNAs in development of chemotherapy resistance. Consistent with this, the result of TargetScan Release in our research indicated that DNMT1 acted as a potential target gene for miR-148b in its mRNA 3′UTR. Further protein expression assay showed that DNMT1 was up-regulated in A549/DDP, importantly, its expression was regulated by over-expression or down-regulation of miR-148b in A549/DDP or A549.

## Conclusions

In summary, this study has shown that the development of drug-resistant NSCLC cells is associated with the deregulation of miR-148b expression. Additionally, we have shown that expression of miR-148b is contradictory relationship with DNMT1 expression in the A549/DDP cells. Furthermore, the silence of DNMT1 expression in the A549/DDP cells reversed miR-148b-induced increases sensitivity of the drug-resistant cancer cells to cisplatin. Targeting the expressed miR-148b or its regulated gene may offer novel therapeutic approaches for the treatment of NSCLC.
